# The impact of cryopreservation on bone marrow-derived mesenchymal stem cells: a systematic review

**DOI:** 10.1186/s12967-019-02136-7

**Published:** 2019-11-29

**Authors:** Soukaina Bahsoun, Karen Coopman, Elizabeth C. Akam

**Affiliations:** 1 School of Sport, Exercise and Health Sciences, Loughborough University, Loughborough, Leicestershire LE11 3TU UK; 2 Centre for Biological Engineering, Loughborough University, Loughborough, Leicestershire LE11 3TU UK

**Keywords:** Bone-marrow derived mesenchymal stem cells, Cell therapy, Cryopreservation, Mesenchymal stem cells, Tissue culture, Systematic review

## Abstract

Mesenchymal stem cells (MSCs) represent an invaluable asset for the field of cell therapy. Human Bone marrow-derived MSCs (hBM-MSCs) are one of the most commonly used cell types in clinical trials. They are currently being studied and tested for the treatment of a wide range of diseases and conditions. The future availability of MSCs therapies to the public will require a robust and reliable delivery process. Cryopreservation represents the gold standard in cell storage and transportation, but its effect on BM-MSCs is still not well established. A systematic review was conducted to evaluate the impact of cryopreservation on BM-MSCs and to attempt to uncover the reasons behind some of the controversial results reported in the literature. Forty-one in vitro studies were analysed, and their results organised according to the cell attributes they assess. It was concluded that cryopreservation does not affect BM-MSCs morphology, surface marker expression, differentiation or proliferation potential. However, mixed results exist regarding the effect on colony forming ability and the effects on viability, attachment and migration, genomic stability and paracrine function are undefined mainly due to the huge variabilities governing the cryopreservation process as a whole and to the lack of standardised assays.

## Background

Bone marrow non-hematopoietic stem cells represent a fraction of the bone marrow cell population. They may arise from the constituents of the bone marrow structure and they can differentiate into mesenchymal tissues such as adipose, cartilage and bone. Bone marrow non-hematopoietic stem cells were first mentioned by Julius Cohnheim in 1867 and later cultured and characterized by Freidenstein et al. in the 1970s [1–4]. Friedenstein demonstrated that bone marrow non-hematopoietic stem can be selected by adherence to culture flask and exhibit the following characteristics: fibroblast morphology, colony-forming ability and in vitro proliferation and differentiation potentials [5]; all of which were indicative of ‘stemness’ properties [6]. With that said, it must be noted that within the scientific community, there is still an ongoing discussion about the true nature of these cells. Two names propagated for these cells “Stromal Stem Cells” [7] and “Mesenchymal Stem Cells” [8, 9].

The then newly discovered source of stem cells has attracted great interest in medical research. In addition to the characteristics listed above, isolating mesenchymal stem cells from bone marrow was surrounded with minimal ethical issues and could substitute embryonic stem cells [6]. Therefore, hBM-MSCs became the subject of intense research and in 1995 the first autologous intravenous infusion of these cells in cancer patients was performed [10]. Later, MSCs have been shown to have widespread immunomodulatory effects [11] as well as an angiogenic induction ability [12]. Taken together these characteristics enlarged the scope of application of hMSC-based therapies. As of April 2019, a search on the U.S. National Library of Medicine (ClinicalTrials.gov) using the term ‘bone marrow mesenchymal stem cells’ retrieved 368 clinical trials aiming to treat conditions like stroke, graft versus host disease, osteoarthritis, crohn’s disease, ischemic heart disease and multiple sclerosis.

The future availability of cell therapies to the public will be dependent on easy and quick logistics as well as robust and reliable delivery process. Abazari et al. [13] suggested that if cell therapies “cannot be delivered clinically and logistically then their benefit is irrelevant”. Cryopreservation remains the cell therapy industry “standard” for biopreservation [14] as well as the primary option of storage for hMSC-based products [15]. In fact, cryostorage has evolved from being a marginal process in the cell therapy manufacturing process to become a tool widening the availability of stem cell therapy in particular and regenerative medicine in general. However, despite its evolving role, cryobiology is lagging behind the speed at which the cell therapy industry is growing.

Cryopreservation is particularly crucial for a successful cell therapy for various reasons. It facilitates cell transport, it enables the generation of cell banks with indefinite shelf-life thus ensuring off-the-shelf steady supply, access and availability and it gives time for quality control testing and in vitro assays [14, 16, 17]. In addition, cryostoring therapeutic doses of cells in hospitals and clinics could make cell therapy a treatment choice for many diseases and conditions including acute conditions [18]. Furthermore, cryopreserved cells are ideal for sequential treatments such as the case of chronic heart failure or ischemic heart disease to ensure the consistency of the treatment [19]. Banking cells is also an appropriate option from an economical and a regulatory aspect [20]. The logistics of administration of MSC in many immunotherapy trials were simply described as cryopreserving cells, thawing them when needed and administering them within a couple of hours. This scenario would only be feasible if thawed cells preserved their viability, safety and potency [20].

Cryopreservation of cells is associated with several injuries; physical and molecular. A controversy still exists about the efficacy of fresh cells versus cryopreserved and whether viability implies functionality [21]. In early MSC-based clinical trials, using cryopreserved cells was hypothesised to be the source of failure [21]. In addition, the variability in the outcome of MSC-based clinical trials was proposed to mainly be due to the functional alterations that the freeze–thaw process provokes in MSCs rather than the freezing method itself [17].

Human Bone marrow-derived MSCs (hBM-MSCs) are the most commonly used source of MSCs in clinical trials [22] and have been deployed across 17 European centres manufacturing MSCs [23]. The effects of cryopreservation on this type of cells are not well defined. The aim of this review is to assess whether rigorous data exist regarding the impact of the freeze–thawing process on BM-MSCs phenotypic and functional traits. To our knowledge, this is the first review to factor numerous aspects of the freezing process (freezing solution composition, the freezing protocol, the duration of storage, the concentration of cells at freezing, the passage number at freezing as well as the thawing method) in one analysis, for studies conducted over about 20 years. Such detailed analysis may allow firm conclusions to be drawn regarding BM-MSCs performance after the freeze-thawing process as well as help uncover possible reasons behind some of the controversial existing results and highlight areas which require further investigation.

## Methods

**Fig. 1:**
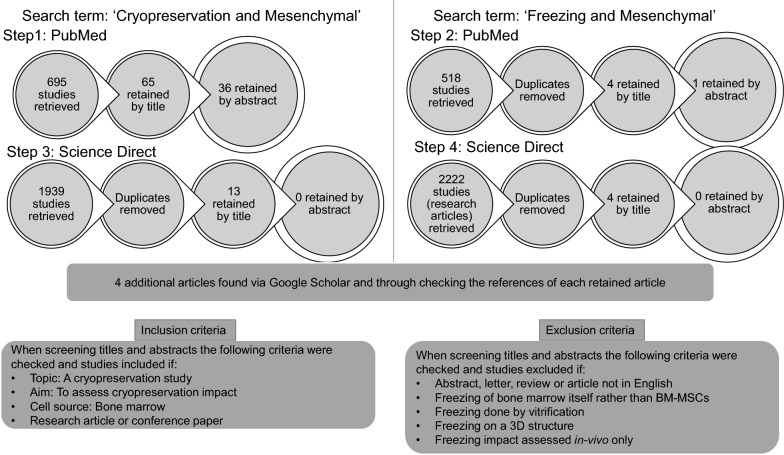
Schematic representation of the Bone-marrow derived mesenchymal stem cell cryopreservation search strategy. Diagram of the current systematic search analysis. Studies of bone-marrow derived mesenchymal stem cells aligned to cryopreservation and/or freezing were identified using a combination of two search terms ‘cryopreservation mesenchymal’ and ‘freezing mesenchymal’ using PubMed, Science direct and Google scholar. The output of each search was scanned at the title level, then at the abstract level and articles were retained when meeting eligibility criteria, both inclusion and exclusion (see boxes titled inclusion criteria and exclusion criteria in this figure). In specific, for the term ‘cryopreservation and mesenchymal’ in PubMed, 695 studies were retrieved. By checking the titles against the eligibility criteria, only 65 studies were retained. The abstracts of these 65 articles were then read and checked against the eligibility criteria and only 36 of the 65 articles were retained. For the subsequent searches, these steps of retaining and eliminating articles were followed but preceded by eliminating duplicates i.e. articles which appeared in previous searches

The inclusion criteria for this review were: Articles or conference papers assessing the impact of cryopreservation by slow freezing on BM-MSCs in suspension. There was no restriction on the species from which cells were derived. Studies where bone marrow itself was frozen, where freezing of BM-MSCs was done by vitrification or using a 3D structure and where cryopreservation impact was only assessed in vivo, were excluded. A systematic literature search was conducted using PubMed, Science direct and Google Scholar (last search performed April 2019). Two combinations of search terms were used ‘cryopreservation mesenchymal’ and ‘freezing mesenchymal’. The output of each search was first scanned for the relevance of title. Articles were excluded if the topic is unrelated or when an eligibility criterion is not met. The retained articles were then screened for the relevance of abstracts (and in few cases materials and methods) and retained when meeting all the eligibility criteria (Fig. 1). From the 41 retained studies, information regarding the freezing solution composition, the freezing protocol, the duration of storage, the concentration of cells at freezing, the passage number at freezing as well as the thawing method was extracted and tabulated. Next, studies were grouped in tables according to the “hMSC checklist” proposed in [24]. Cell surface marker expression, differentiation potential, proliferation and growth, attachment and migration potential, genomic stability and paracrine function were examined. In addition, post-thaw viability and morphology information was also collated because they are primary evaluators of cryopreservation.

## Results

### Species, freezing and thawing methods

**Fig. 2:**
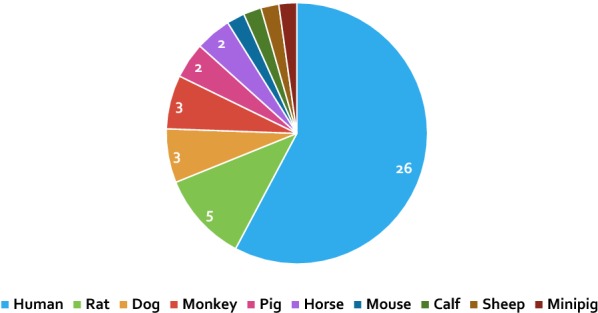
Pie chart showing the number of studies per species. The numbers of studies are presented on the diagram in Arabic numerals unless it is only a single study i.e. where no number is presented this species only represents a single included study. Of note, 3 of the 41 studies appear more than once because they have used more than one species, hence the total number of studies as it appears in this pie chart is 45

As shown in Fig. 1 41 studies met the inclusion criteria. MSCs were isolated from the bone marrow of 10 different species which included human (26 studies), rat (5 studies), monkey (3 studies), dog (3 studies), horse (2 studies), pig (2 studies), minipig (1 study), mouse (1 study), calf (1 study) and sheep (1 study) (Fig. 2).

Across the retained studies, various freezing media formulations were used. 20% of studies (17% human) used commercially available freezing solution such as CELLBANKER and CRYOSTOR while the rest used “in-lab” homemade formulations. 66% of studies (41% human) used or tested various amounts of serum in the freezing media with the serum principally being animal-derived, 20% of studies (12% human) froze cells in serum-free media while 5% used freezing media containing plasma or human platelet lysates (all of which are human studies). For 17% of the studies, not enough information was included about FBS content and/or about the composition of the freezing medium (12% human). Of the serum-free studies, one study assessed the efficiency of Sericin as a substitute to FBS [25]. Across all of the studies, 13 assessed the freezing in xeno-free media. More than 90% of studies used dimethylsulfoxide (DMSO) at a concentration ranging from 1 to 20% with 10% being the most commonly used. Carboxylated poly-l-lysine (COOH-PLL) was investigated as a cryoprotectant to replace DMSO [26] and hydroxyethyl starch was added to freezing solution as a strategy to reduce the percentage of DMSO [27]. Two studies tested various freezing solutions containing polyethylene glycol (PEG), trehalose and 1,2 propanediol in order to develop a well-defined, serum-free and reduced-DMSO freezing solution [28, 29]. Only two studies utilized strategies to prevent post-thaw apoptosis through the addition of Rho-associated kinase inhibitor [30] and Caspase inhibitor z-VAD-fmk [31] in the freezing media.

Concerning freezing protocols, two procedures prevail. The first involves incubating the cells at a freezing rate of − 1 °C/min in a − 80 °C freezer for several hours (up to 24 h) then moving the cells to liquid nitrogen (LN_2_). The second is based on a two to seven-step sequential freezing process using a programmable freezing device to freeze the cells prior to − 150 °C freezer or LN_2_ storage. Four studies reported whether cells were stored in liquid phase [32] or vapour phase [30, 33, 34] of LN_2_. Five studies stored the cells at − 80 °C [26, 35–38] and 1 study stored the cells at − 70 °C [39].

Seven studies (3 human and 4 animal) did not specify the passage number at which cells were frozen. Six studies (3 human and 3 animal) used cells at passage 1, one study (monkey) used cells at passage 9 and the rest (20 human and 6 animal) used cells at passages ranging from 1 to 6. The concentration of cells at freezing was very variable ranging from 1 * 10^5^ to 1 * 10^7^ cells/mL with 1 * 10^6^ cells/mL being the most frequently used (17 studies; 9 human and 8 animal). There was only one study in which human-derived cells were frozen in cryopreservation bags at a concentration of 1.8 * 10^8^ [40]. There was a huge variation regarding the duration of storage of cells in the frozen state; the shortest period being 1 h [30] and the longest 10 or more years [37].

Seven studies (human) did not include information about their thawing protocols. Two studies (1 human and 1 animal) just mentioned ‘quickly thawed’ [20, 39], one in α-MEM [41], one at room temperature [37] and one in a 37 °C incubator [42]. For all the rest (16 human and 13 animal), there was some consistency; cells were typically thawed at 37 °C, most likely in a water bath with or without gentle agitation for 1–4 min.

### Post-thaw assessment

**Fig. 3:**
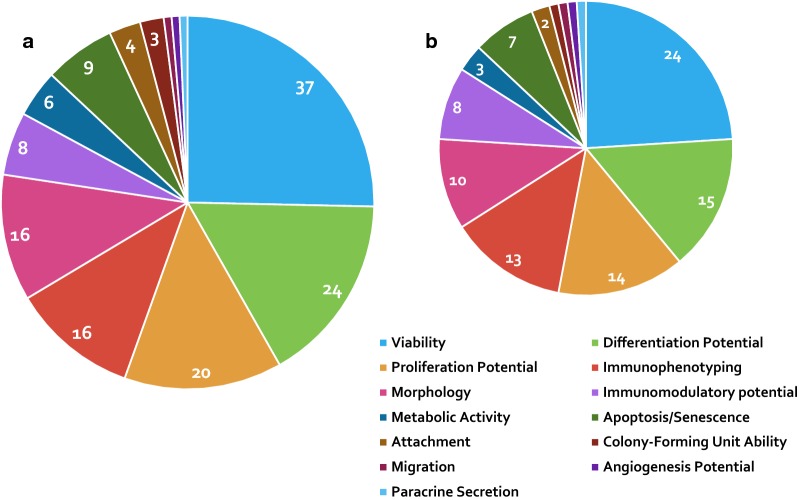
**a** Pie chart showing the proportion of the retained studies assessing different cellular attributes in all species.** b** Pie chart illustrating the proportion of human retained studies assessing different cellular attributes. It is supported by Additional file 1 which aggregates and delineates which studies undertook which analyses in a tabular form [arranged by species: human (chronologically and then alphabetically) and animals from most to least frequent species (chronologically and then alphabetically)]. Additional file 1 is a grid identifying which cell attributes each of the forty-one studies assessed. Of note each of the 41 studies may appear more than once depending on the attributes they assessed

Presented in Fig. 3 are how many of the forty-one retained studies assessed different cellular attributes, it shows that viability and differentiation potential are the only attributes assessed in over half of the retained studies.

### Viability

Post-thaw viability was the most assessed cell attribute (37 studies). Table 1 lists the studies which assessed viability immediately post-thaw (37 studies) or after a period of post-thaw culture, which was monitored in five studies (post-thaw time point from 4 h to 3 passages). Three main methods for viability assessment were used; trypan blue exclusion, flow cytometry and fluorescent microscopy. The immediately post-thaw viability varied from about 50% to 100% which is noteworthy. Sixteen studies reported no change in viability immediately after thawing while 10 studies reported significantly lower viability. In some studies where a new freezing formulation was tested, viability was compared to 10% DMSO which is still considered the gold standard for freezing BM-MSCs. The timing of viability measure is crucial due to the induction of apoptotic events some time post-thaw [43]. As mentioned above only five studies assessed the long-term effect (up to 3 passages post-thaw of freezing) with two reporting lower viability and three reporting no effect. It was also noted that variability in the level of viable cells within the same study differed when using different methods of measurement such as trypan blue exclusion compared to flow cytometry [44, 45].

**Table 1:** Experimental studies assessing viability immediately post-thaw or after a period of post-thaw culture

Study	Species	Method of freezing	Concentration at freezing	Method of thawing	Passage number at freezing	Results post-thaw	Method of assessment
Human							
Bruder et al. [91]	Human	FBS with 10% DMSO in LN2 (24 h)	NA	NA	NA	Cell recovery after thawing was above 95%	Trypan blue exclusion
Hirose et al. [41]	Human	Cell Banker storage medium, cells cryopreserved at − 150 °C (NA)	5 * 10^5^ cells/mL	Cells were thawed in MEM-α supplemented with 15% FBS	P1	Immediately post-thaw viability was retained post-thaw at about 98%	Fluorescent microscopy: live/dead viability assay kit
Kotobuki et al. [35]	Human	Cell Banker medium, cryopreserved at − 80 °C (NA)	5 * 10^5^ cells/mL	NA	P1	Immediately post-thaw viability was retained post-thaw at above 90%	NucleoCounter (ChemoMetec)
Kotobuki et al. [92]	Human	Cell Banker storage medium (ready-to-use storage medium), then cells stored sequentially: 10 min at 4 °C, 1 h at − 30 °C, 2–3 days at − 80 °C then long-term storage at − 152 °C (0.3–33.6 months)	5 * 10^5^ cells/mL	NA	P1	Immediately post-thaw viability ranged from 71.9 to 100% with average viability about 90%	NucleoCounter (ChemoMetec)
Xiang et al. [93]	Human	30% serum-containing α-MEM with 10% DMSO, 4 °C for 10 min then cooled to − 80 °C at 1 °C/min in a controlled-rate freezer then LN2 (12 months)	1 * 10^5^ cells/mL	Thawed in a 37 °C water bath by shaking lightly for 1 or 2 min	P3	Immediately post-thaw viability ranged from 84.6 to 100%	Flow cytometry—fluorescein diacetate, PI
Zhao et al. [94]	Human (with chronic myeloid leukaemia)	IMDM with 40% FCS and 10% DMSO at 4 °C, beaker with methanol in − 70 °C freezer for 24 h then LN2 (3 or 6 months or 1 year)	1 * 10^6^ cells/mL	37 °C water bath for 2–4 min	P2–3	Immediately post-thaw viability was retained post-thaw at about 90%	Trypan blue exclusion
Heng [30]	Human	Culture medium with 10% DMSO and 0, 10 or 100 microM of Rho-associate kinase (ROCK) inhibitor Y-27632, cooling to − 80 °C for 2 h, then vapour phase of LN2 (1 h)	1.17 * 10^5^ cells/mL	Thawed in a 37 °C water bath	P5	Immediately post-thaw viability dropped to a range about 91.3% to 89.4%; No effect of Y-27632 immediately post-thaw but there was an increase in viability at 24 h post-thaw	Trypan blue exclusion
Liu et al. [28]	Human	13 different freezing media tested with various combinations of different concentrations of serum, DMSO, PEG, trehalose and 1.2-Propanediol, equilibration of cells with freezing media at 4 °C for 10 min, − 80 °C overnight then LN2 (min. 1 week)	1 * 10^6^ cells/mL	Thawed in a 37 °C water bath, shaking gently for 2 min	N/A	A freezing solution composed of 7.5% c(v/v) DMSO, 2.5% (w/v) PEG, 2% bovine serum albumin gave comparable viability (about 82.9%) to 10% DMSO (about 82.7%)	Flow cytometry–PI
Doan et al. [95]	Human	DMEM/F12 with 10% DMSO, incubation, 4 °C for 10 min, − 20 °C for 1 h, − 80 °C for 1 day then LN2 (1 year)	1 * 10^6^ cells/mL	In a water bath at 37 °C	P3	Immediately post-thaw viability was retained post-thaw at 72.95%	Cell Viability Analyzer (Beckman Counter, USA)
François et al. [45]	Human	α-MEM with 30% FBS and 5% DMSO, − 80 °C for 24 h then LN2 (1 week)	NA	NA	Early passage	Immediately post-thaw viability dropped to ≤ 60% (Annexin V/PI) and > 80% (Trypan blue); At 4 h post-thaw viability was between 44 and 61%; viability increased after post-thaw culture	Trypan blue exclusion
Ginis et al. [50]	Human	CryoStor-2, CryoStor-5, CryoStor-10 containing 2%, 5% and 10% DMSO respectively or conventional freezing medium (90% growth medium with 10% FCS, 30% bovine serum albumin and 10% DMSO), pre-cooling on ice for 10 min, slowly cooled to − 5 °C, blast of chilling to − 25 °C, quick return to − 5 °C, cooling to − 60 °C at a rate of 1 °C/min, cooling to − 196 °C at a rate of − 25 °C using programmable cell freezer then LN2 (about 1 month or 5 months)	1 * 10^6^ cells/mL	Thawed fast in a 37 °C water bath with gentle agitation	P2–4	Immediately post-thaw viability after 1-month freezing was about 91.7% and 95.6% and 95.4% for CryoStor-2, CryoStor-5, CryoStor-10 respectively; Immediately post-thaw viability after 5-month freezing was about 72% and 80% for CryoStor-5 and CryoStor-10 respectively	Fluorescence uptake: calcein-AM, ethidium homo-dimer-1
Mamidi et al. [33]	Human	90% FBS with 10% DMSO, programmable slow freezing unit then vapour phase of LN2 (long-term storage)	3 * 10^6^ cells/2 mL vial	Thawed in a 37 °C water bath, shaking gently for 1–2 min	P3 and then characterized at P4–6 (with another freezing at passage 4)	Viability was about 80% upon thawing then > 95% after subsequent plating (3 passages post-thaw)	Trypan blue exclusion; flow cytometry—7-AAD
Matsumura et al. [26]	Human	COOH-PLLs 7.5% (w/w) at pH of 7.4 OR 10% DMSO in DMEM without FBS, − 80 °C freezer (1 week or 24 months)	1 * 10^6^ cells/mL	Thawed in a 37 °C water bath with gentle shaking	P3–5	Cryopreservation for one week with PLL (0.5–0.8) did not affect the viability at 0 h and 6 h post-thaw; Cryopreservation for 24 months with PLL (0.65) provides protection comparable to 10% DMSO	Trypan blue exclusion
Chinnadurai et al. [20]	Human	Freezing media, − 80 °C then LN2 (NA)	5 * 10^6^ cells/mL	Quickly thawed (1–2 min)	P3–5	Immediately post-thaw viability dropped to about 87% (trypan blue) and 71.5% (flow cytometry)	Trypan blue exclusion; flow cytometry—Annexin V, PI
Holubova et al. [69]	Human	60% α-MEM medium with 30% pHPL and 10% DMSO, programmable controlled rate freezer at rate 1 °C/min to − 80 °C then LN2 (1,3,6,7 and 8 months)	1 * 10^6^ cells/mL	NA	P3	Immediately post-thaw viability is 70–90%	Flow cytometry—7-AAD staining
Moll et al. [38]	Human	4 °C human blood type AB plasma containing 10% DMSO, frozen to − 80 °C using rate-controlled cell freezing device (NA)	1–2 * 10^6^ cells/mL	NA	P2–4	Viability reduced twofold by cryopreservation when exposed to human serum (cell count and PI incorporation)	Cell counter and analyser system (CASY-TT); flow cytometry–Annexin V, PI
Verdanova et al. [25]	Human	15 different freezing solutions containing various concentrations of DMSO (0, 1, 5, 10 and 100%) in the presence or absence of sericin at 1 or 5%, cooling to − 80 °C at a rate 1 °C/min in a CoolCell container then LN2 (72 h)	1.4 * 10^5^ cells/mL	In a 37 °C water bath as quickly as possible	P1–3	Highest viability (24 h post-thaw) was obtained using standard freezing medium (10% DMSO and 25% FBS in culture medium); Viability of cells (24 h post-thaw) frozen in culture medium containing 10% DMSO and 1% sericin was not significantly different from standard freezing medium	Fluorescent microscopy—DAPI
Al-Saqi et al. [66]	Human	10% DMSO in Mesencult-XF or STEM-CELLBANKER at 4 °C, cryovials on ice then moved to − 80 °C with a cooling rate − 1 °C/min for 24 h then then LN2 (NA)	0.5–1 * 10^6^ cells/mL	Thawed in a 37 °C water bath for 1 or 2 min	P3	No difference in viability immediately post-thaw between two freezing media; CELLBANKER (85.6%) and 10% DMSO (86%); No significant difference in viability between non-cryopreserved and cryopreserved using both media	Fluorescence-based live/dead assay immediately post-thaw; flow cytometry—PI (two passages post-thaw)
Luetzkendorf et al. [40]	Human	5% human albumin and 10% DMSO, automatized process in a programmable freezer then LN2 (21–51 days)	1.8 * 10^8^ in cryopreservation bags	Thawed at	P3–4	Immediately post-thaw viability was retained at > 90% viability using both methods for 4 donors out of 5	Trypan blue exclusion; flow cytometry: 7-AAD
Pollock et al. [67]	Human	60% plasmalyte A, 20% of 25% HAS and 20% DMSO (final concentration of DMSO was 10% by volume), controlled rate freezer then LN2 (30–45 days)	1–10 * 10^6^ cells/mL	Thawed quickly in a 37 °C	P1–6	Immediately post-thaw viability was retained at > 80% for almost all samples	Fluorescent microscopy—Acridine orange, PI
Chinnadurai et al. [68]	Human	IFNɣ, caspase inhibitor Z-VAD-FMK or 3-methyl adenine pre-licensing 48 h prior to cryopreservation, 5% human serum albumin, 5%, 20%, 40%, 90% hPL in aMEM with 10% DMSO OR CryoSOfree DMSO-free cryopreservation medium, cooling rate 1 °C/min then step-down freezing using a 7-step program in CryoMed controlled-rate freezer then LN2 (NA)	5–10 * 10^6^ cells/mL	In a 37 °C water bath for 1 min	P2–6	The addition of various concentrations of human platelet lysate did not significantly enhance MSC recovery and viability; IFNɣ pre-licensing prior to cryopreservation enhances thawed MSC survival	Trypan blue exclusion; flow cytometry—7-AAD
Gramlich et al. [18]	Human	CryoStor CS5 media, − 80 °C for 90 min then vapour phase of LN2 (7–30 days)	1 * 10^6^ cells per mL	In a 37 °C water bath	P3–5	Immediately post-thaw viability was retained at > 95% (viability only marginally reduced after thawing)	TUNEL staining; Fluorescent microscopy—Hoechst, PI
Lechanteur et al. [34]	Human	40% PBS + 40% of HSA solution (20%) + 20% DMSO added under agitation at 4 °C, automated cryofreezer with a 9-step program to − 160 °C then vapour phase of LN2 (NA)	2 * 10^6^ cells/mL	Freezing bag is protected in sterile plastic bag and thawed in a 37 °C water bath for a few min	P3	Immediately post-thaw viability ranged from about 50% to 90% with about 14% decrease in viability	Trypan blue exclusion
Yuan et al. [52]	Human (BM-MSC engineered to express TRAIL)	5% DMSO, 30% FBS in alpha-MEM OR human albumin with 0.5–20% DMSO, isopropanol freezing box overnight in, − 80 °C freezer then LN2 (1–3 weeks)	1 * 10^6^ cells/mL or 5 * 10^6^ cells/mL or 10 * 10^6^ cells/mL	In a water bath at 37 °C with gentle shake for 2 min	P5	Significantly reduced immediately post-thaw viability with 0% DMSO (5.16%); Immediately post-thaw viability increased with increased DMSO% in the freezing; 15% and 20% DMSO gave reduced viability (about 70.6% and 64.1% respectively) immediately post-thaw solution up to 10%; at 5% DMSO same viability obtained for different cell concentrations	Flow cytometry—Annexin V, DAPI
Other species							
Carvalho et al. [44]	Rat	DMEM with 10% FBS and 5% DMSO, cells incubate at room temperature for 15 min then vials cooled at 3 °C/min, 5 °C/min, 10 °C/min during 15, 45, 10 min respectively until − 80 °C using programmable freezing device then LN2 (1 month)	1 * 10^7^ cells/mL	Thawed in a 37 °C water bath with constant gentle shaking	Frozen down after 4 weeks in culture	Immediately post-thaw viability dropped to about 90.58% (trypan blue) and 66.25% (flow cytometry)	Trypan blue; flow cytometry—Annexin V, 7-AAD
Liu et al. [29]	Rat, mouse and calf	14 different freezing solutions tested with various combinations of different concentrations of serum, DMSO, PEG, trehalose and 1.2-Propanediol, equilibration for 15 min at 4 °C, − 80 °C overnight then LN2 (min. 1 week)	1 * 10^6^ cells/mL	Thawed in a 37 °C water bath with gentle shaking for 2 min	NA	There were variations between species with respect to cell viability—Mouse MSCs were more robust than rat and bovine MSCs; Reduced DMSO (5%) with 2% PEG, 3% trehalose and 2% albumin gave higher immediately post-thaw viability (91.5% [mouse]) to 10% DMSO (75.3% [mouse])	Trypan blue exclusion
Naaldijk et al. [27]	Rat	Cryoprotectant consisted of hydroxyethyl starches of different mean molecular weights [MW = 109, 209, 309, 409, 509, 609 kDa] and/or DMSO, then cells were frozen according to one of seven different freezing protocols (NA)	1 * 10^5^ cells/0.5 mL	Thawed in a 37 °C water bath	P1–3	Immediately post-thaw viability was approximately 85%; viability after 3 days of thawing was lower	Trypan blue exclusion
Davies et al. [42]	Rat	10% DMSO in 90% FBS, then vials incubated for 1 h at 4 °C, 2 h at − 20 °C, overnight at − 80 °C then LN2 (NA)	1 * 10^6^ cells/mL	Thawing in a 37 °C RS Galaxy S + incubator for about 5 min	P1	Immediately post-thaw viability was retained post-thaw at > 90%; But lower viability was obtained after in vitro expansion of cryopreserved cells	Trypan blue exclusion
Renzi et al. [31]	Sheep, horse and rat	13 different freezing media tested with various combinations of different concentrations of FBS, DMSO, Trehalose, hydroxyethyl starch, bovine serum albumin and Caspase inhibitor z-VAD-fmk, 4 °C for 60 min, gradual reduction of temperature − 1 °C/min to − 40 °C, − 10 °C/min to − 70 °C in a controlled rate freezer then vapour phase of LN2 (5 days)	1 * 10^6^ cells/mL	Thawed in a 37 °C water bath	P4	No DMSO or low DMSO gave very poor viability; The best viability was obtained when using FBS with 10% DMSO	Trypan blue exclusion (evaluated at 0, 24 and 48 h post-thaw)
Li et al. [96]	Dog	DMEM with 10% FBS and 10% DMSO, 4 °C for 1 h, − 20 °C for 2 h, − 80 °C for 10.5 h then LN2 (1 month)	1 * 10^6^ cells/mL	Thawed at 37 °C	P4	Immediately post-thaw viability was retained post-thaw at 90.1%	Trypan blue exclusion
Zhu et al. [46]	Dog	DMEM containing 10% FBS and 10% DMSO, 4 °C for 1 h, − 20 °C for 2 h, − 80 °C for 10.5 h then LN2 (3 years)	1 * 10^6^ cells/mL	Thawed in at 37 °C	P4	No significant difference in cell viability	Trypan blue exclusion
Edamura et al. [36]	Dog	Cryoprotectant solution with or without 10% DMSO and 10% FBS, biofreezing vessel at − 80 °C in a freezer (7 days)	1 * 10^6^ cells/mL	Thawed in a 37 °C water bath for 1 min	P1	DMSO and FBS-free freezing gave higher viability (about 99%); DMSO and FBS containing freezing media gave lower viability (about 89.7%)	Trypan blue exclusion
Nitsch et al. [97]	Monkey	Freezing medium containing 0,1,5,10 or 15% DMSO (v/v), controlled rate freezer using an optimised freezing rate then − 150 °C freezer (1 week)	1 * 10^6^ cells/mL	In a 37 °C water bath	P9	Immediately post-thaw viability was about 80% for the different DMSO concentrations; Highest viability 24 h post-thaw for cells frozen with 5 or 10% DMSO	Trypan blue exclusion
Lauterboeck et al. [49]	Monkey	Three different freezing solutions tested (2 of them xeno-free) containing different concentrations of DMEM, DMSO and/or FBS, methylcellulose, poloxamer-188, α-tocopherol, cell suspension equilibrated for 10, 30 or 60 min then placed in controlled rate freezer using one-step freezing protocol or two-step freezing protocol then − 150 °C (at least 24 h)	1 * 10^6^ cells/mL	In a 37 °C water bath for 90 s	NA	Viability maintained after thawing	Automatic cell counter
Ock and Rho [51]	Pig	ADMEM solution supplemented with 10% FBS and 1% penicillin–streptomycin with 40%, 20% or 10% DMSO, controlled rate programmable freezing device at − 1 °C/min from 25 °C to − 80 °C then then LN2 (< 1 month)	2 * 10^6^ cells/mL	In a 37 °C water bath for 1 min	P5	There was a significant difference between fresh and cells cryopreserved with 10% (about 77.6%) or 20% DMSO (about 67%); No significant difference between fresh and cells cryopreserved with 5% DMSO (about 83.9%)	Trypan blue exclusion
Romanek et al. [98]	Pig (BM-MSC treated with a high hydrostatic pressure (HHP) before freezing)	10% DMSO, 2 h at − 20 °C then LN2 (up to 4 weeks)	NA	37 °C water bath with gentle shaking	NA	Significant difference between cells treated with HHP and control immediately post-thaw (about 75.2%–81.7%); No difference in viability at 8 days post-thaw (about 81.6%–82.1%)	Trypan blue exclusion
Mitchell et al. [32]	Horse	Six different freezing solutions tested (20% serum [autologous equine serum, commercial equine serum or FBS], 10% DMSO and 70% media OR 95% serum and 5% DMSO), − 80 °C freezer for 24 h then liquid phase of LN2 (2–5 days)	10 * 10^6^ cells/mL	In a 35 °C water bath with gentle agitation	P3–6	Immediately post-thaw viability was retained at about 80–90% regardless of the cryopreservation formulation	Flow cytometry—Fluorescein diacetate, PI

Details on the experimental cryopreservation processes taken by different research groups. This table aims to provide the individual freezing protocols outlined in the extracted papers alongside the concentration and passage of cells at the point of cryopreservation and the process of thawing

### Morphology

Table 2 summarises the studies which assessed post-thaw cell morphology. This attribute was mainly assessed using microscopy. Irrespective of all the variables considered in this data analysis and the time post-thaw at which cell morphology was checked, 13 of the 16 studies agree that cryopreservation itself has no effect on post-thaw cell morphology. The addition of Rho-associate kinase (ROCK) inhibitor Y-27632 was reported to give BM-MSCs a web-like appearance which indicated some neuronal differentiation [30]. In addition, several cell shapes were observed at day 2 and day 5 post-thaw [46] and cell shrinkage was detected using flow cytometry [38].

**Table 2:** Bone-marrow derived mesenchymal stem cell studies assessing post-thaw cell morphology

Study	Species	Results post-thaw	Method of assessment
Human			
Kotobuki et al. [92]	Human	No effect on morphology	Microscopy (fluorescent/phase contrast)
Haack-Sorensen et al. [19]	Human	No effect on morphology	NA
Xiang et al. [93]	Human	No effect on morphology	Microscopy (light) at cell confluency post-thaw
Zhao et al. [94]	BM-MSC (human with chronic myeloid leukemia)	No effect on morphology	NA
Heng [30]	Human	The addition of Y-27632 altered the morphology of the cells (web-like appearance)	NA
Liu et al. [28]	Human	No effect on morphology	Microscopy (fluorescent)
Doan et al. [95]	Human	No effect on morphology	Microscopy (light) 7 days post
Mamidi et al. [33]	Human	No effect on morphology	NA
Moll et al. [38]	Human	Effect of cryopreservation seen on forward scatter but not side scatter when exposed to human serum	Microscopy; cell counter and analyser system (CASY-TT); Flow cytometry
Al-Saqi et al. [66]	Human	No effect on morphology	Microscopy (light) two passages post
Other species			
Liu et al. [29]	Rat, mouse and calf	No effect on morphology	Microscopy (light)
Naaldijk et al. [27]	Rat	No effect on morphology	Microscopy (light)
Davies et al. [42]	Rat	No effect on morphology	Microscopy (phase contrast)
Zhu et al. [46]	Dog	Cells had several shapes such as long fusiform, polygon and astroid	Checked at days 2 and 5 after thawing NA
Edamura et al. [36]	Dog	No effect on morphology	Microscopy (light)
Mitchell et al. [32]	Horse	No effect on morphology	Microscopy (light) (24 and 72 h post)

The key results on bone-marrow derived mesenchymal stem cell morphology are presented in this table. For further details on the cryopreservation experimental details refer to either Table 1 or Additional file 2 which provide the individual freezing protocols outlined in the extracted papers alongside the concentration and passage of cells at the point of cryopreservation and the process of thawing

### Immunophenotyping

Marker expression is one of the International Society for Cellular Therapy (ISCT) criteria for defining MSCs [47] so it is of real importance to check BM-MSCs phenotype before freezing and/or after thawing. Table 3 lists the studies which assessed BM-MSCs marker expression post-thaw. Despite its importance, less than half of the 41 studies retained assessed post-thaw marker expression retention and despite all the variables taken into consideration in this investigation, there was a consensus regarding the methodology used (flow cytometry) and the results; cryopreservation does not affect BM-MSCs immunophenotype.

**Table 3:** Bone-marrow derived Mesenchymal Stem Cell studies evaluating surface marker expression post-thaw

Study	Species	Results post-thaw	Method of assessment
Human			
Kotobuki et al. [92]	Human	No difference	Flow cytometry
Haack-Sorensen et al. [19]	Human	No difference	Flow cytometry
Xiang et al. [93]	Human	No difference	Fluorescent sorting at passage 1, 5, 10 and 15 post-thaw
Zhao et al. [94]	Human (with chronic myeloid leukaemia)	No difference	Flow cytometry
Doan et al. [95]	Human	No difference	Flow cytometry
Ginis et al. [50]	Human	No difference except lower expression of CD9	Flow cytometry
Mamidi et al. [33]	Human	No difference	Flow cytometry
Matsumura et al. [26]	Human	No difference	Flow cytometry
Holubova et al. [69]	Human	No difference	Flow cytometry
Moll et al. [38]	Human	No difference	Flow cytometry
Al-Saqi et al. [66]	Human	No difference	Flow cytometry
Luetzkendorf et al. [40]	Human	No difference	Flow cytometry
Yuan et al. [52]	Human (BM-MSC engineered to express TRAIL)	No difference	Flow cytometry
Other species			
Naaldijk et al. [27]	Rat	No difference	Flow cytometry
Davies et al. [42]	Rat	No change in the expression of CD29 and CD73; Increase in the expression of CD90, CD44 and CD105	Flow cytometry for CD29 and CD90; RT-qPCR for CD44, CD105 and CD73
Ock and Rho [51]	Pig	No difference	Flow cytometry

The main results on bone-marrow derived mesenchymal stem cell surface marker expression are presented in this table. For further details on the cryopreservation experimental details refer to either Table 1 or Additional file 2 which provide the individual freezing protocols outlined in the extracted papers alongside the concentration and passage of cells at the point of cryopreservation and the process of thawing

### Differentiation potential

Tri-lineage differentiation (adipogenic, osteogenic and chondrogenic) is another criterion listed by the ISCT guide for defining MSCs [47]. Hence, more than half of the studies [24] assessed BM-MSCs post-thaw differentiation potential and these are listed in Table 4. Osteogenesis is the most frequently assessed differentiation pathway (20 studies) qualitatively through Alizarin red staining and/or quantitatively through measurement of alkaline phosphatase activity. There was an agreement among 18 studies that cryopreservation did not affect BM-MSCs osteogeneic potential. One study reported lower osteogenesis [39] and one reported improved osteogenesis post-thaw [48]. Adipogenesis was next in terms of frequency of testing using Oil Red O staining as a qualitative assessment and no effect of cryopreservation was observed in 12 studies. Only one study provided a quantitative assessment of adipogenesis and it was the only one reporting lower differentiation level [49].

**Table 4:** Published experimental studies detailing BM-MSCs post-thaw differentiation potential

Study	Species	Results post-thaw	Method of assessment
Human			
Bruder et al. [91]	Human	No effect on osteogenic differentiation ability	Cell re-plated for one passage post-thaw then re-plated and incubated with osteogenic supplements; quantification of alkaline phosphatase activity
Hirose et al. [41]	Human	No effect on osteogenic differentiation ability	Incubation with osteogenic media for 25 days; quantitative fluorescence analysis of calcein uptake
Kotobuki et al. [35]	Human	No effect on osteogenic differentiation ability	Incubation with osteogenic medium for 2 weeks; calcium and alkaline phosphatase activity staining
Kotobuki et al. [92]	Human	No effect on osteogenic differentiation ability	Incubation in osteogenic media for 2 weeks; quantification of alkaline phosphatase activity and calcein uptake
Xiang et al. [93]	Human	No effect on adipogenic or neuro genic differentiation ability	Cells at P15 post-thaw incubated in adipogenesis medium for 12 days; Oil Red O staining
			Cells at P15 post-thaw incubated in neurogenesis induction medium for 1 or 6 days; fluorescent staining and RT-qPCR
Zhao et al. [94]	BM-MSC (human with chronic myeloid leukaemia)	No effect on differentiation ability	Incubation in osteogenic medium for 21 days; Von Kossa staining and RT-qPCR
			Incubation in adipogenic media for 14 days; Oil Red staining and RT-qPCR
			Incubation in neurogenic medium; Immunocytochemistry and western blotting for NF, GFAP and GalC
			Endothelial differentiation for 2 weeks; immunohistochemical and western blotting for CD31 and vWF
Liu et al. [28]	Human	Serum-free reduced-DMSO freezing solution gives comparable differentiation to 10% DMSO	Incubation in osteogenic or adipogenic media for 2 weeks, and chondrogenic media for 3 weeks
Doan et al. [95]	Human	No effect on adipogenic differentiation ability	Incubation in adipogenic medium for 2–3 weeks; Oil Red staining
Ginis et al. [50]	Human	No effect on osteogenic differentiation ability	Incubation with osteogenic media; quantification of alkaline phosphatase activity on days 7 and 14 after incubation as well as flow cytometry analysis at day 14 after incubation of alkaline phosphatase surface expression
			Quantification of calcium deposition at day 21 after incubation
Mamidi et al. [33]	Human	No effect on tri-lineage differentiation ability	Incubation in osteogenic differentiation media for 3 weeks; Alizarin red staining
			Incubation in adipogenic differentiation media for 3 weeks; Oil Red O staining
			Incubation in chondrogenic differentiation media for 3 weeks; Alcian blue staining
Matsumura et al. [26]	Human	No effect on tri-lineage differentiation ability	Incubation in osteogenic differentiation media for 14 days; Alizarin red staining and alkaline phosphatase activity
			Incubation in adipogenic differentiation media for 14 days; Oil Red O staining and GPDN activity
			Incubation in chondrogenic differentiation media for 14 days; Alcian blue staining
Kumazawa et al. [37]	Human	No effect on adipogenic or osteogenic differentiation ability	Incubation in osteogenic medium for 1, 2, and 3 weeks then alkaline phosphatase activity, calcium levels, alizarin red staining and RT-qPCR
			Incubation in adipogenic medium for 1, 2, and 3 weeks; Oil Red O staining
Luetzkendorf et al. [40]	Human	No effect on adipogenic and osteogenic differentiation ability	Incubation in diff media until morphological signs of differentiation were visible (10–15 days)
Lechanteur et al. [34]	Human	No effect on differentiation ability	NA
Yuan et al. [52]	Human (BM-MSC engineered to express TRAIL)	No effect on tri-lineage differentiation ability	Differentiation procedures performed using StemPro differentiation kits according to manufacturer’s instructions
Other species			
Liu et al. [29]	Rat, mouse and calf	No effect on adipogenic or osteogenic differentiation ability	Incubation with adipogenic media for 2 weeks; Oil Red O staining and alkaline phosphatase activity expression staining with BCIP/NBT
			Incubation with adipogenic or osteogenic media for 2 weeks then Oil Red O staining and alkaline phosphatase activity expression staining with BCIP/NBT
Naaldijk et al. [27]	Rat	In general, no difference in differentiation was observed (qualitative observation); Quantification of ALP: ALP activity is lower at ‘high’ (> 5%) levels of DMSO compared to solutions with a higher HES 450 content	Incubation with osteogenesis, adipogenesis and chondrogenic media for 2 weeks
			Quantification using alkaline phosphatase assay
Li et al. [96]	Dog	No effect on osteogenic differentiation ability	Incubation in osteogenic media for 5, 10 and 15 days; alkaline phosphatase activity measurement
			Incubation in osteogenic media for 21 days; number of mineralized nodules determined
Zhu et al. [46]	Dog	No effect on osteogenic differentiation ability	Incubation with osteogenic media (21 days); measurement of alkaline phosphatase activity at 5, 10 and 15 days and Von Kossa staining and nodules counting at day 21
Edamura et al. [36]	Dog	No effect on neurogenic differentiation ability	Incubation with neurogenic media for 6 h then RT-qPCR
Tokumoto et al. [48]	Monkey	Cryopreserved cells had a slightly higher ALP enzyme activity than non-cryopreserved cells	Incubation with osteogenic media for 4, 8 and 12 days; quantification of alkaline phosphatase enzyme activity
Nitsch et al. [97]	Monkey	No effect on adipogenic or osteogenic differentiation ability	Incubation with adipogenic media for 5 weeks; oil-red O staining
			Incubation with osteogenic medium for 21 days; Von Kossa staining
Lauterboeck et al. [49]	Monkey	Significant decrease in oil droplet formation	Incubation with adipogenic medium for 20 days; Oil Red O staining
		No difference in osteogenic differentiation ability	Incubation with osteogenic induction medium for 3 weeks; Alizarin red staining
Heino et al. [39]	Minipig	Cells lost their osteogenic differentiation potential	Cells incubated in osteogenic medium; stained for alkaline phosphatase activity (tested 9 days post-thaw)

Results on bone-marrow derived mesenchymal stem cell tri-linage are presented in this table. For further details on the cryopreservation experimental details refer to either Table 1 or Additional file 2 which provide the individual freezing protocols outlined in the extracted papers alongside the concentration and passage of cells at the point of cryopreservation and the process of thawing

Chondrogenesis presented as the least studied tri-linage differentiation pathway. Only five studies differentiated thawed BM-MSCs into chondrocytes with a qualitative assessment made via Alcian Blue staining. It was concluded that cryopreserved cells did not lose the ability to chondrogenic differentiation (of note thawed BM-MSCs were also able to commit to neuronal and endothelial lineages).

### Proliferation potential

Table 5 lists the 20 studies which examined post-thaw BM-MSCs proliferation potential. Various methods were used to determine proliferation rate such as population doublings and DNA quantification. The majority of results agree that cryopreservation does not affect post-thaw BM-MSCs proliferation potential, nonetheless lower proliferation rate was obtained by two studies [34, 39] and higher proliferation rate was obtained by one study [50]. Colony-forming unit ability, a traditional measure of BM-MSCs proliferation, was assessed by three studies with mixed results [25, 32, 51].

**Table 5:** Experimental studies evaluating post-thaw BM-MSCs proliferation potential

Study	Species	Results post-thaw	Method of assessment
Human			
Bruder et al. [91]	Human	No effect on proliferation	Cell re-plated for one passage post-thaw; crystal violet dye-binding method
Haack-Sorensen et al. [19]	Human	No effect on proliferation	PKH26-GL cell linker kit
Xiang et al. [93]	Human	No effect on proliferation	Growth curves
Zhao et al. [94]	Human (with chronic myeloid leukaemia)	No effect on proliferation	Cell count and cell-doubling time
Doan et al. [95]	Human	No effect on proliferation	NA
Ginis et al. [50]	Human	Proliferation of cryopreserved cells after 1 or 5 months of storage was higher than non-cryopreserved cells	Calcein-AM staining (at day 1, 4, 7 and 14 after post-thaw plating)
Mamidi et al. [33]	Human	No effect on proliferation	Population doublings, cumulative population doublings and population doubling time
Matsumura et al. [26]	Human	No effect on proliferation	Cell count; population doubling time (24 h, 48 h, 72 h and 96 h post-thaw)
Holubova et al. [69]	Human	No effect on proliferation	Cell count
Al-Saqi et al. [66]	Human	No significant difference in population doubling time but cells cryopreserved in DMSO had longer population doubling time compared to fresh	Population doubling (first and second passage post-thaw)
Luetzkendorf et al. [40]	Human	No effect on proliferation	Population doublings; Population doubling time
Pollock et al. [67]	Human	Population doublings decreased with increasing pre-freeze passage number	Population doublings
Lechanteur et al. [34]	Human	Very low recovery until day 4 then a slight increase indicating re-proliferation	Cell count (0–5 days after thawing)
Yuan et al. [52]	Human (BM-MSC engineered to express TRAIL)	No effect on proliferation	XTT assay
Other species			
Edamura et al. [36]	Dog	DMSO and FBS-free freezing resulted in similar proliferative capacity as non-cryopreserved; DMSO and FBS containing freezing media gave lower proliferative capacity	Cell count (2, 4, 6,8, 10 and 12 days post-thaw)
Tokumoto et al. [48]	Monkey	No effect on proliferation	DNA quantification at 4, 8 and 12 days
Lauterboeck et al. [49]	Monkey	No effect on proliferation	Population doubling time
Heino et al. [39]	Minipig	Two to sixfolds decrease in the proliferative capacity of cells	Population doublings
Romanek et al. [98]	Pig (BM-MSC treated with a high hydrostatic pressure (HHP) before freezing)	Cells treated with HHP showed better proliferation rate	Cell count
Mitchell et al. [32]	Horse	No effect on proliferation	Cell staining with CellTrace label
Colony-forming unit ability			
Human			
Verdanova et al. [25]	Human	Best number of colonies obtained when cells were frozen with 5% DMSO with 5% sericin in culture medium	Cells seeded 60 cm Petri dishes for 2 weeks, Crystal Violet stained and colonies counted (light microscope)
Other species			
Ock and Rho, [51]	Pig	All cryopreserved cells showed significantly lower numbers of colonies compared to fresh; Lower DMSO produced higher number of colonies	Cells seeded in 6-well plates for 2 weeks, 4% Giemsa stained and colonies counted (light microscope)
Mitchell et al. [32]	Horse	No effect on colony-forming unit ability	Cells seeded in 10 cm plates for 1 week, Crystal Violet stained and colonies counted (light microscope)

The key results on bone-marrow derived mesenchymal stem cell proliferation are presented in this table. For further details on the cryopreservation experimental details refer to either Table 1 or Additional file 2 which provide the individual freezing protocols outlined in the extracted papers alongside the concentration and passage of cells at the point of cryopreservation and the process of thawing

### Metabolic activity

Table 6 lists the six studies which examined post-thaw BM-MSCS metabolic activity. Three methods were equally used to determine cell metabolic activity; AlamarBlue, Presoblue and MTT (3-(4,5-dimethylthiazol-2-yl)-2,5-diphenyltetrazolium) reduction-based assays. From the data collated it could be seen that two-thirds of experiments performed reported impaired metabolic activity post-thaw.

**Table 6:** Bone-marrow derived mesenchymal stem cell studies evaluating post-thaw metabolic activity

Study	Species	Results post-thaw	Method of assessment
Human			
Liu et al. [28]	Human	Reduced-DMSO freezing solution gives comparable metabolic activity to 10% DMSO	AlamarBlue assay
Chinnadurai et al. [20]	Human	No reduction in metabolic fitness	Calcium uptake; PrestoBlue reduction
Chinnadurai et al. [68]	Human	The addition of various concentrations of hPL (human platelet lysate) did not significantly enhance MSC metabolic activity	PrestoBlue reduction
Other species			
Liu et al. [29]	Rat, mouse and calf	In general, non-cryopreserved cells showed higher overall metabolic activities than the cryopreserved; Reduced DMSO (5%) with 2% PEG, 3% trehalose and 2% albumin give superior results to 10% DMSO	AlamarBlue assay
Nitsch et al. [97]	Monkey	Lower metabolic activity for cryopreserved cells compared with fresh; Enhnaced levels of metabolic activity obtained for 5% and 10% DSMO levels	MTT assay (24 h, 48, 72 and 96 h post-thaw)
Lauterboeck et al. [49]	Monkey	Cells’ metabolic activity was impaired up until 48 h post-thaw; Partial recovery at 72 h and full recovery observed at 96 h	MTT assay (24 h, 48, 72 and 96 h post-thaw)

The key results on bone-marrow derived mesenchymal stem cell metabolic activity after cryopreservation are presented in this table. For further details on the cryopreservation experimental details refer to either Table 1 or Additional file 2 which provide the individual freezing protocols outlined in the extracted papers alongside the concentration and passage of cells at the point of cryopreservation and the process of thawing

### Apoptosis and senescence levels

Typically assessed using flow cytometry, the induction of apoptosis is evident when considering the six studies entered in Table 7. However, cryopreservation does not seem to induce senescence (refer Table 7) although more studies are needed to draw a firm conclusion.

**Table 7:** The induction of apoptosis in post-thaw BM-MSCs

Study	Species	Results post-thaw	Method of assessment
Apoptosis			
Human			
Liu et al. [28]	Human	Serum-free reduced-DMSO freezing solution gives comparable apoptotic percentage to 10% DMSO	Flow cytometry
Ginis et al. [50]	Human	Lower percentage of apoptotic cells obtained with Annexin V and Hoechst staining compared to caspase 3 assay: using caspase 3, the percentage of apoptotic cells was between 13 and 17% for CryStor media compared to 3% for conventional freezing media	Flow cytometry—Annexin V, Hoechst, Caspase 3 activity
Chinnadurai et al. [20]	Human	Higher percentage of apoptotic cells in cryopreserved MSC than live MSC	Flow cytometry
Moll et al. [38]	Human	Apoptosis increased by cryopreservation when exposed to human serum	Flow cytometry—Annexin V, PI staining
Other species			
Ock and Rho [51]	Pig	Bak and Bcl2 gene expression in cryopreserved cells was higher than fresh at 3 h post-thaw: Bak and Bcl2 gene expression in cryopreserved cells was comparable to fresh after culturing thawed cells up to 90% confluence: Bcl2 antigen expression level was comparable to fresh after culturing thawed cells up to 90% confluence	RT-qPCR for Bak and Bcl2: Flow cytometry; Bcl2 antigen
Romanek et al. [98]	Pig (BM-MSC treated with HHP before freezing)	No significant difference between control (without HHP) and cells subjected to HHP pre-freeze	Flow cytometry—Annexin V: Fluorescence microscopy
Senescence			
Human			
Mamidi et al. [33]	Human	No difference in the level of senescent cells	Β-galactosidase assay
Al-Saqi et al. [66]	Human	There were signs of senescence (but could be due to culture medium rather than cryopreservation medium)	Β-galactosidase assay (analysed 2 passages after cryopreservation)
Pollock et al. [67]	Human	Immediate pre-freeze senescence levels show similar trends but higher levels compared to pre-freeze At 48 h post-thaw, level of senescent cells dropped significantly comparing to immediately post-thaw	Beta-glo assay

The key results on bone-marrow derived mesenchymal stem cell apoptotic activity post-thaw are presented in this table. For further details on the cryopreservation experimental details refer to either Table 1 or Additional file 2 which provide the individual freezing protocols outlined in the extracted papers alongside the concentration and passage of cells at the point of cryopreservation and the process of thawing

### Attachment and migration

Only four studies assessed BM-MSCs attachment ability post-thaw, and these are recorded in Table 8. This table indicates that frozen cells have lower adherence capability post-thaw. Only one study assessed post-thaw cell migration [52]. It concluded that cryopreservation has no effect on post-thaw cell migration ability.

**Table 8:** Bone-marrow derived Mesenchymal Stem Cell studies evaluating cellular attachment post-thaw

Study	Species	Results post-thaw	Method of assessment
Attachment			
Human			
Heng [30]	Human	Level of adherent cells was 39.8 ± 0.9%; increased by approx. 10% with Y-27632	MTT assay performed 24 h post-thawing
Chinnadurai et al. [20]	Human	40% reduction in adhesion to fibronectin; 80% reduction in adhesion to endothelial cells; No reduction in the surface expression of adhesion molecules	After 2 h in static and 1 h in vascular flow conditions using microscopy (light); Flow cytometry for adhesion molecules
Other species			
Li et al. [96]	Dog	Decreased adhesion capacity post-thaw; recovery of adhesion capacity after culturing for several passages	Adherent cell count (hemo-cytometer) at 4, 8, 12 and 24 h post-thaw
Tokumoto et al. [48]	Monkey	Limited influence of cryopreservation on cell adhesion capabilities	Adherent cell count (hemo-cytometer)
Migration			
Human			
Yuan et al. [52]	Human (BM-MSC engineered to express TRAIL)	No effect on migration potential	Trans-well plates

The effects of cryopreservation on bone-marrow derived mesenchymal stem cell attachment are presented in this table. For further details on the cryopreservation experimental details refer to either Table 1 or Additional file 2 which provide the individual freezing protocols outlined in the extracted papers alongside the concentration and passage of cells at the point of cryopreservation and the process of thawing

### Paracrine function

Paracrine function is related to two main MSCs activities namely immunomodulation and angiogenesis. In total, 10 studies (Refer Table 9) assessed BM-MSCs paracrine function with immunomodulation being the most frequently assessed with eight studies. The results of these studies are equally balanced with four of them reporting no effect of cryopreservation on BM-MSCs post-thaw immunomodulatory potential and four reporting an impaired potential. Angiogenesis potential and secretion of growth factors were only assessed by one study each with no effect of cryopreservation reported.

**Table 9:** Published experimental studies detailing BM-MSCs post-thaw paracrine function

Study	Species	Results post-thaw	Method of assessment
Immunomodulatory potential			
Human			
Zhao et al. [94]	Human (with chronic myeloid leukaemia)	No effect on immunomodulatory potential	Mixed leukocyte reaction inc. T-cell proliferation
François et al. [45]	Human	Impaired inhibition of proliferation of activated T cells; low IDO protein expression in response to INF-γ stimulation; up-regulation of heat shock proteins	T-cell proliferation assay (CD3/CD28); Western blot IDO; RT-qPCR IDO, CCL2, IL-6
Holubova et al. [69]	Human	No effect on immunomodulatory potential	T-cell proliferation (PHA)
Moll et al. [38]	Human	Impaired immunomodulatory properties	RT-qPCR IDO, IL-6; Western blot IDO; Instant blood mediated inflammatory reaction (IBMIR)
Luetzkendorf et al. [40]	Human	No effect on immunomodulatory potential	Co-culture with PBMC; T-cell proliferation (PHA)
Chinnadurai et al. [68]	Human	Freeze-thawing attenuates immunosuppressive properties of human MSC independent of freezing methods or freezing media; Thawed MSC can suppress T-cell proliferation in the absence of cell contact; IFNɣ pre-licensing prior to cryopreservation enhances thawed MSC’s immunosuppressive properties	Co-culture with PBMC; T-cell proliferation (CD3/CD28 & SEB); RT-qPCR IDO, Hsp
Gramlich et al. [18]	Human	No effect on immunomodulatory potential	Co-culture with PBMC; T-cell proliferation (CD3/CD28); IDO activity assay (kynurenine)
Lechanteur et al. [34]	Human	Impaired immunomodulatory properties	Co-culture with PBMC; T-cell proliferation (CD3/CD28); IDO activity assay (kynurenine)
Angiogenesis potential			
Human			
Haack-Sorensen et al. [19]	Human	No effect on the capacity of MSC to differentiate into endothelial cells; Retained VEGF responsiveness	In vitro angiogenesis; RT-qPCR, KDR, vWF, INSIG
Growth factor secretion			
Human			
Gramlich et al. [18]	Human	Small changes in growth factor secretion between fresh and cryopreserved cells	Human antibody-mediated growth factor array

Summary of the effects of cryopreservation on bone-marrow derived mesenchymal stem cell paracrine function are presented in this table. For further details on the cryopreservation experimental details refer to either Table 1 or Additional file 2 which provide the individual freezing protocols outlined in the extracted papers alongside the concentration and passage of cells at the point of cryopreservation and the process of thawing

## Discussion

A recent analysis of MSC-based clinical trials showed that although no safety concerns surround MSC infusion, the translation from bench to bedside is still confronted by what the authors called the ‘Achilles heel’; donor heterogeneity, ex vivo expansion, immunogenicity and cryopreservation [53]. There is no doubt that cryopreservation is essential for MSC therapy translation, both autologous and allogeneic, and is still one the limitations to be addressed.

Cryopreservation by slow freezing can cause two types of cell damage; physical and molecular. Physical injuries were the first to be identified and include ice nucleation, solution effects, osmotic shock, cold shock as well as cryoprotectant toxicity [14]. Molecular injuries encompass the effect of cryopreservation on gene expression, protein levels, cell functionality, the induction of stress response as well as post-thaw epigenetic changes [54, 55].

In the case of MSCs, studying the effect of molecular injuries and how to mitigate them is a twofold problem. Firstly, investigating molecular injuries is still a developing branch of cryobiology. In fact, immediately post-thaw cell viability has always been the most assessed cell attribute in cryopreservation studies. However, it has been shown that signs of cellular damage may take some time to manifest (cryopreservation-induced delayed-onset cell death [54]). Leading to viability and functional losses which are compounded by a lack of detection and reporting in immediate post-thaw analysis. Approaches to tackle molecular injuries, intracellular-like freezing solutions and anti-apoptotic compounds, can be deployed yet research in this area is still at an early stage [54].

Secondly, establishing and standardising potency markers and assays to characterise MSCs is still a challenge [24]. In fact, MSCs possess variability in their gene expression profiles, differentiation and expansion potential and phenotype depending on tissue origin, cell isolation and expansion procedures [56] as well as donor characteristics [57]. In 2006, the ISCT published a guideline on minimal criteria to define MSC; plastic adherence, expression of certain surface markers and lack of others and tri-lineage differentiation [47]. In 2013, these criteria were expanded to include a fourth parameter, quantification of MSC immune functional potency [58]. In 2016, the society suggested “a matrix assay approach: quantitative RNA analysis of selected gene products; flow cytometry analysis of functionally relevant surface markers and protein-based assay of the secretome” in order to fulfil the fourth criterion [59].

Currently, there are no standard markers or potency assays to typify MSCs or evaluate their post-thaw potency despite much discussion within the scientific community [24, 60]. Therefore, research laboratories follow differing protocols which makes data evaluation complex. As both research areas (freezing molecular injuries and MSC characterisation) develop so will the methodology of evaluating MSC cryopreservation.

There are profound variabilities in the whole cryopreservation process from freezing media formulation, method of freezing and thawing and duration of storage to passage number and cell concentration at freezing. However, despite all these variabilities and all the species included, there was evidence showing that four BM-MSCs attributes are stable and unaffected by the stresses imposed by freezing and thawing and these are: cell morphology, marker expression, proliferation potential and tri-lineage differentiation capability (although chondrogenesis was only assessed by five independent studies). The four other attributes viability, attachment and migration, genomic stability and paracrine function were governed by either conflicting results or by low assessment frequency.

All studies have employed a strategy to evade freezing and thawing physical damage. Freezing BM-MSCs at a slow cooling rate (at least at the start of the cooling process) and thawing at a high rate was followed by almost all the studies in this review. In terms of media formulation, DMSO remains the most commonly used cryoprotectant to protect BM-MSCs (used by 90% of studies analysed). However, DMSO is associated with adverse effects such as cardiac side effects [61, 62] and severe neurotoxicity [63, 64] when infused in patients. Consequently, reducing or eliminating DMSO, depending on clinical outcome, may become a requisite.

Mixing other potential cryoprotectants with DMSO at percentages < 10% indicates that this traditionally held protectant and percentage can both be altered [26–28]. This is an interesting result which indicates that eliminating DMSO from freezing solution is a viable option and is worthy of further evaluation based on a wider post-thaw MSC functional characteristics. Another important aspect of MSC cryopreservation is the fact that FBS is commonly added to freezing solutions for its benefits to stabilise the cell membrane and adjust cell osmotic pressure [65]. The main issue in using FBS is that it is animal-derived and may cause a xenogenic reaction if infused in patients [65]. Cryopreserving hBM-MSCs in xeno-free media was assessed across 13 studies [18, 26, 34, 38, 40, 48–50, 52, 66–69]. Verdanova et al. reported using Sericin (a protein derived from silkworm cocoon) as an FBS substitute for preserving human BM-MSCs [25]. The possibility of overlapping xeno-free and low or no DMSO exists in one study where monkey BM-MSCs were successfully frozen in xeno-free solution composed of methylcellulose, poloxamer, α-tocopherol and only 2.5% DMSO [49]. Such a freezing solution would be ideal because it is not only xeno-free but also contains a very low DMSO concentration.

Cell concentration at freezing and the duration of storage are not well studied, and it is not well documented if it plays a role in the performance of the end-product. The only study which tested cell viability after freezing at three different cell concentrations found no significant variation [52]. In regard to duration of storage, of note is the study where BM-MSCs were stored for more than 10 years without losing multipotency [37]. This could well be a suggestion that once cells enter a quiescent state, its duration is not of great significance.

The evaluation of the cryopreservation process is certainly an existing challenge. Our understanding of the biology of MSCs is evolving and so are the possibilities of the application of these cells in the medical field. Yet, as stated recently “Significant challenge remains the development of a relevant potency assay” [34]. These assays must be quick, easy and should not require trained personnel if they are to be used to release each cell batch in a clinical setting and if they are to fit with operation theatre logistics (thawing, testing and infusing within a couple of hours). In theory, potency assays could be therapy-specific and must indicate cell functionality; in other words, “mechanism(s) of action” [70]. How much these assays correlate with the in vivo niche is also of great importance. In addition, “the assay should be able to differentiate between sufficiently potent and sub-potent batches, a (semi-)quantitative assay is required” [56]. This thawing-infusing scenario would be realistic only if cryopreservation methods have improved to give an optimal product immediately after thawing.

Cell morphology (shape and size) can give indication on cell’s health as well as whether they have committed to differentiate or not. The absence of change in BM-MSCs morphology after cryopreservation can indicate that the freeze-thaw process does not cause differentiation or change in cell phenotype. This conclusion is further evidenced by the absence of effect of cryopreservation on BM-MSCs marker expression post-thaw.

No such firm conclusion can be drawn when it comes to viability. This is of real importance given that viability is one of the release criteria for cell therapies. In fact, viability has and will always be the primary indicator on cryopreservation success. It is an easy, cheap and fast measurement but is has some limitations. The various methods used across labs to measure viability and the lack of a unified reporting structure makes it hard to compare results. Although ≥ 90% viability for fresh MSC product and ≥ 70% viability for cryopreserved MSC product are generally considered the benchmark for clinical application [57], different labs report viability maintenance or loss of viability based on comparison with pre-freeze viability or on comparison with freezing with 10% DMSO (refer Table 1). In addition, the common measurement time-point, only immediately post-thaw could be misleading due to the late manifestation of the effect of current cryopreservation protocols on cells.

In fact, the initiation of apoptotic events is evident according to the data in Table 7. Despite the importance of this cryopreservation-related cell death, it is surprising how limited the investigation of this molecular pathway in thawed BM-MSCs is and the strategies to reduce it are. Only two studies utilized strategies to prevent post-thaw apoptosis (molecular injury). The addition of Rho-associated kinase inhibitor Y-27632 in the freezing medium and in the post-thaw culture medium did not improve hBM-MSCs viability immediately but recorded enhanced recoveries at 24-h post-thaw [30]. However, the addition of Caspase inhibitor z-VAD-fmk in the freezing media did not prove to be beneficial for equine, ovine and rodent BM-MSCs as assessed by viability immediately, at 24 and 48 h post-thaw [31].

An expert workshop on preservation and stability of cell therapy products was held in May 2015 [71]. Assessing post-thaw viability was one of the topics discussed and the limitations/conflicts mentioned above were identified. The group advised that assessing cell viability post-thaw should “go beyond simple enumeration of cell numbers to facilitate a greater understanding of the cell system in question”. In addition, the group realised the need for more advanced and accurate methods to assess cell viability that can be linked to cell function [71].

Interestingly, impaired cell metabolism can be closely associated with apoptotic pathways through the Bcl-2 family proteins which initiates apoptosis in metabolically stressed cells through utilization of autophagy as a nutrient source before ultimately undergoing necrosis [72, 73]. From the data presented in Table 6 some impairment of BM-MSCs metabolic activity post-thaw is evident. Therefore, a link between defective metabolic activity in thawed BM-MSCs and a higher level of apoptosis post-thaw could be postulated. This may indicate new pathways to mitigate against these post-thaw phenomena.

More research is needed if cells are to be thawed and immediately infused in patients. Infusing apoptotic cells will hinder MSCs therapeutic benefits. Moreover, it is vital that therapeutic cells emerge potent from freezing to be capable to survive in a damaged tissue where they will encounter a hostile environment with mechanical, hypoxia and nutritional stresses, the host immune response and inflammation. These factors are known to cause a huge loss in MSC viability after transplantation as well as poor engraftment [74]. According to Table 8, frozen cells have lower adherence capability. One study has tried to examine this post-thaw phenomena in more detail lending evidence to a disruption of F-actin polymerization rather than shedding of surface adhesion receptors [20]. Advancing our knowledge in this area will help manufacture clinical MSCs with improved regenerative engraftment, although the concept of MSCs proliferating, differentiating and engrafting in host tissue as their primary therapeutic modality has been recently challenged [75].

Generally, it can be said that cryopreservation does not affect BM-MSCs proliferative capacity (Table 5). However, intriguing evidence is presented by Ginis et al. [50] on the potential cell selection that the cryopreservation process may enforce. In this study, BM-MSCs post-thaw proliferation potential was higher than that at pre-freeze. The authors justified their results as “selection of stronger cells after cryopreservation” and suggested that their results should “alarm a scientific community”. This same theory has also been mentioned by Baust et al. [76] who stated, “unstudied but of concern is the potential for the preservation process to select for increased resistance to preservation stresses”.

From Table 5, it can be concluded that there is a strong agreement that cryopreservation does not affect BM-MSCs differentiation potential. This conclusion is of value to fulfil one of the 2006 ISCT criteria for MSCs. However, differentiation potential may become less important for cell therapy. For example, in heart disease, MSCs’ initial mechanism of regeneration was outlined as differentiation into cardiomyocytes and incorporation into the host tissue. Recently, this outline has been updated to shed a light on a more effective regeneration mechanism and that is their associated paracrine signalling [75]. In fact, paracrine signalling is not confined to cardiac regeneration but is now generally considered as the MSCs main mode of action. Caplan [77] suggested that it is “time to change the name” of MSCs to “Medicinal Signalling Cells” in order to better describe the secretion of trophic factors. According to Gonzalez et al. [78] “80% of the therapeutic effect of stem cells is attributed to paracrine actions”.

The MSCs secretome is composed of growth factors and cytokines either soluble or engulfed in exosomes and/or vesicles [75, 79, 80]. MSC paracrine signalling is described as exerting plethora of effects including induction of angiogenesis, regulation of immune response and inflammation, modulation of cell differentiation and proliferation, extracellular matrix formation, neuroprotective and neurotrophic effects, anti-apoptotic, anti-tumour and anti-microbial activities [81, 82]. Yet despite such a compelling list of activities no comprehensive evaluation of the MSC secretome, and or its cell-free utility, has been conducted [83]. Data on the BM-MSCs secretome upon thawing is very limited and so is the data on angiogenic potential. There are only two studies which concluded that no effect of cryopreservation was observed (Table 9).

At an injury site, MSC contribute to the creation of an anti-inflammatory environment by suppressing the activation and proliferation of pro-inflammatory cells and promoting anti-inflammatory cells [84]. This modulation of both the innate and adaptive immune system can be accomplished in two ways: via cell–cell interaction and cell–cell communication through an array of soluble factors including indoleamine 2,3-dioxygenase (IDO) and/or extracellular vesicles [85]. These characteristics were the main contributors in raising the MSC profile as therapeutically valuable. As discussed above, MSC immune function has now become an essential indicator on MSC function. In view of this, it is expected that more studies on MSC immunomodulatory properties, and assays to evaluate them will emerge. Recently, Chinnadurai et al. [86] tested BM-MSCs potency using a matrix approach and concluded that cryopreservation negatively affects cells’ secretome with T cell proliferation. According to Table 9, eight studies assessed BM-MSCs immune function post-thaw with a balance of four concluding damaging effect and four concluding no effect.

## Conclusion

**Fig. 4:**
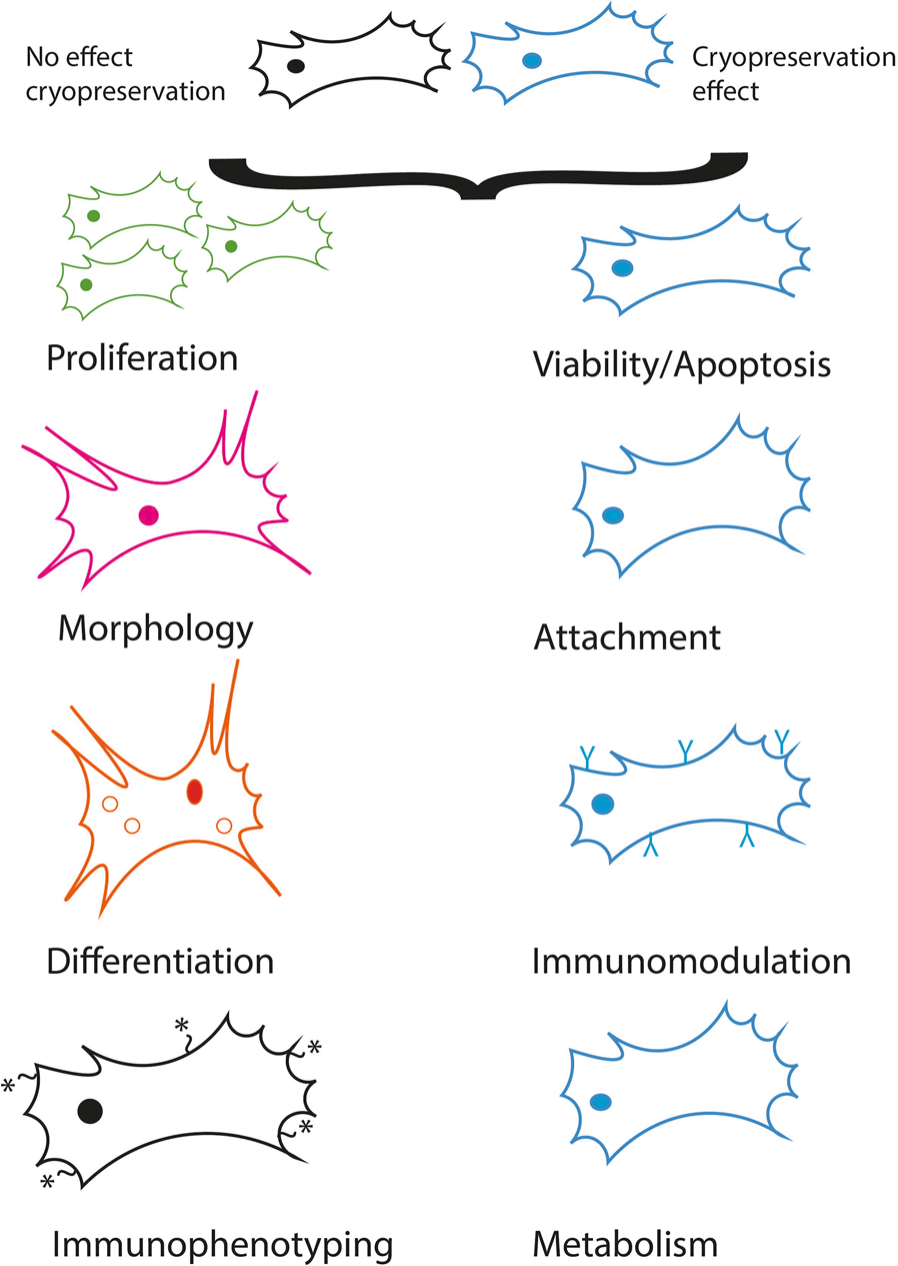
Effects of cryopreservation on BM-MSC in terms of cellular attributes and function. From the systematic analysis performed, cryopreservation appears to influence viability and apoptosis, cellular attachment, immunomodulation and metabolism (cell schematics shown in blue on right-hand side). Whereas, no common significant effects mediated by cryopreservation have been documented in proliferation, morphology, differentiation or immunophenotyping (cell schematics given in green, red, orange and black on the left-hand side)

This systematic review has highlighted areas of agreement and deviation that currently exist around BM-MSCs properties and functions after cryopreservation (Fig. 4).

With MSC-based therapies expected to offer treatment choices for several conditions and diseases that are currently incurable and presently no MSC drug yet approved by the FDA the biological community must work towards reproducible and reliable data sets to achieve regulatory accepted drug status. With cryopreservation effects being fully identified and assessed in terms of therapeutic evaluation.

Continuing variance in our scientific approaches facilitates unapproved therapies and stem cell tourism [87, 88]; leading both the FDA and ISCT to urge both caution for individuals and request enhanced rigor, reproducibility and visibility from the scientific community [59, 89, 90]. “Successful new therapies come at a considerable cost that cannot easily be sustained without evaluation and guidance” [89].

This review has been limited to one tissue source and that is bone marrow, yet we know that MSCs can be isolated from almost all tissues especially adipose and umbilical cord [17]. Including more tissue sources was beyond the scope of this review yet it is important to document the impact of cryopreservation on those MSC sources also.

## Supplementary information

**Additional file 1.** Table of cellular attribute data from studies extracted for the systematic review. It is a grid identifying which cell attributes each of the forty-one studies assessed. Of note each of the 41 studies may appear more than once depending on the attributes they assessed. Where a study undertook an assessment of a cellular attribute a cross is placed in the grid. Studies are arranged by species: human [chronologically and then alphabetically] and animals from most to least frequent species [chronologically and then alphabetically]). Column headers: Morphology; Morph. Viability; Via. Immunophenotyping; IP. Differentiation; Diff. Colony forming unit frequency; CFUF. Growth; Total growth. Metabolism; Met. Apoptosis; Apo. Attachment; Attach. Immunomodulation; Immuno. Paracrine; Para. Angiogenesis; Angio. Migration; Migr.

**Additional file 2.** Tabulated information relating to the freezing details extracted from the relevant studies. It shows the details of the individual freezing protocols outlined in the 41 retained studies. The method of freezing is given in detail alongside the species information, the concentration and passage of cells at the point of cryopreservation and the process of thawing. These details are common to the results tables (Tables 1, 2, 3, 4, 5, 6, 7, 8, 9).

## Data Availability

All data generated by this systematic search are included in this published article.

## Abbreviations

7-AAD: 7-aminoactinomycin D
α-MEM: alpha modified eagle medium
ADMEM: advanced Dulbecco’s medium eagle media
COOH-PLLs: carboxylated poly-l-lysine
DMEM: Dulbecco’s medium eagle media
DMEM/F12: 1:1 mixture of Dulbecco’s Medium and Ham’s F-12 medium
DMSO: dimethylsulfoxide
EMEA: DMEM, low glucose with HEPES 1×
FBS: fetal bovine serum
FCS: fetal calf serum
H: hour(s)
hPL: human platelet lysate
IMDM: Iscove’s modified Dulbecco’s medium
INF-γ: interferon-gamma
MEM: modified eagle medium
microM: micromolar
Min: minute(s)
NA: not available
P: passage
PEG: polyethylene glycol
PHA: phytohemagglutinin
pHPL: pooled human platelet lysate
PI: propidium iodide
SEB: Staphylococcal enterotoxin B
