# Supplementary material for: The impact of cryopreservation on bone marrow-derived mesenchymal stem cells: a systematic review
Source: J Transl Med. 2019 Nov 29;17:397. doi: 10.1186/s12967-019-02136-7 (PMC6883667; doi:10.1186/s12967-019-02136-7)
Supplement: Supplementary file 1 — Additional file 1. Table of cellular attribute data from studies extracted for the systematic review. It is a grid identifying which cell attributes each of the forty-one studies assessed. Of note each of the 41 studies may appear more than once depending on the attributes they assessed. Where a study undertook an assessment of a cellular attribute a cross is placed in the grid. Studies are arranged by species: human [chronologically and then alphabetically] and animals from most to least frequent species [chronologically and then alphabetically]). Column headers: Morphology; Morph. Viability; Via. Immunophenotyping; IP. Differentiation; Diff. Colony forming unit frequency; CFUF. Growth; Total growth. Metabolism; Met. Apoptosis; Apo. Attachment; Attach. Immunomodulation; Immuno. Paracrine; Para. Angiogenesis; Angio. Migration; Migr. [file 12967_2019_2136_MOESM1_ESM.docx]

Additional information 1. Table of cellular attribute data from studies extracted for the systematic review

| **Study** | **Morph.** | **Via.** | **IP** | **Diff.** | **CFUF** | **Growth** | **Met.** | **Apo.** | **Attach.** | **Immuno** | **Para.** | **Angio.** | **Migr.** |
| --- | --- | --- | --- | --- | --- | --- | --- | --- | --- | --- | --- | --- | --- |
| **Human** | | | | | | | | | | | | | |
| Bruder, Jaiswal and Haynesworth, 1997 (90) |  | × |  | × |  | × |  |  |  |  |  |  |  |
| Hirose et al., 2004 (41) |  | × |  | × |  |  |  |  |  |  |  |  |  |
| Kotobuki et al., 2004 (35) |  | × |  | × |  |  |  |  |  |  |  |  |  |
| Kotobuki et al., 2005 (91) | × | × | × | × |  |  |  |  |  |  |  |  |  |
| Haack-Sorensen et al., 2007 (19) | × |  | × |  |  | × |  |  |  |  |  | × |  |
| Xiang et al., 2007 (92) | × | × | × | × |  | × |  |  |  |  |  |  |  |
| Zhao et al., 2008 (93) | × | × | × | × |  | × |  |  |  | × |  |  |  |
| Heng, 2009 (30) | × | × |  |  |  |  |  |  | × |  |  |  |  |
| Liu et al., 2010 (28) | × | × |  | × |  |  | × | × |  |  |  |  |  |
| Doan et al., 2012 (96) | × | × | × | × |  | × |  |  |  |  |  |  |  |
| François et al., 2012 (45) |  | × |  |  |  |  |  |  |  | × |  |  |  |
| Ginis, Grinblat and Shirvan, 2012 (50) |  | × | × | × |  | × |  | × |  |  |  |  |  |
| Mamidi et al., 2012 (33) | × | × | × | × |  | × |  | × |  |  |  |  |  |
| Matsumura et al., 2013 (26) |  | × | × | × |  | × |  |  |  |  |  |  |  |
| Chinnadurai et al., 2014 (20) |  | × |  |  |  |  | × | × | × |  |  |  |  |
| Holubova et al., 2014 (68) |  | × | × |  |  | × |  |  |  | × |  |  |  |
| Kumazawa et al., 2014 (37) |  |  |  | × |  |  |  |  |  |  |  |  |  |
| Moll et al., 2014 (38) | x | x | × |  |  |  |  | x |  | × |  |  |  |
| Verdanova, Pytlik and Kalbacova, 2014 (25) |  | × |  |  | × |  |  |  |  |  |  |  |  |
| Al-Saqi et al., 2015 (65) | × | × | × |  |  | × |  | × |  |  |  |  |  |
| Luetzkendorf et al., 2015 (40) |  | × | × | × |  | × |  |  |  | × |  |  |  |
| Pollock et al., 2015 (66) |  | × |  |  |  | × |  | × |  |  |  |  |  |
| Chinnadurai et al., 2016 (67) |  | × |  |  |  |  | × |  |  | × |  |  |  |
| Gramlich et al., 2016 (18) |  | × |  |  |  |  |  |  |  | × | × |  |  |
| Lechanteur et al., 2016 (34) |  | × |  | × |  | × |  |  |  | × |  |  |  |
| Yuan et al., 2016 (51) |  | × | × | × |  | × |  |  |  |  |  |  | × |
| **Other Species** | | | | | | | | | | | | | |
| Carvalho et al., 2008 (44) [rat] |  | × |  |  |  |  |  |  |  |  |  |  |  |
| Liu et al., 2011 (29) [rat, mouse,calf] | × | × |  | × |  |  | × |  |  |  |  |  |  |
| Naaldijk et al., 2012 (27) [rat] | × | × | × | × |  |  |  |  |  |  |  |  |  |
| Davies et al., 2014 (42) [rat] | × | × | × |  |  |  |  |  |  |  |  |  |  |
| Renzi et al., 2012 (31) [sheep, horse,rat] |  | × |  |  |  |  |  |  |  |  |  |  |  |
| Li et al., 2009 (94) [dog] |  | × |  | × |  |  |  |  | × |  |  |  |  |
| Zhu et al., 2013 (46) [dog] | × | × |  | × |  |  |  |  |  |  |  |  |  |
| Edamura et al., 2014 (36) [dog] | × | × |  | × |  | × |  |  |  |  |  |  |  |
| Tokumoto et al., 2008 (48) [monkey] |  |  |  | × |  | × |  |  | × |  |  |  |  |
| Nitsch et al., 2014 (97) [monkey] |  | × |  | × |  |  | × |  |  |  |  |  |  |
| Lauterboeck et al., 2016 (49) [monkey] |  | × |  | × |  | × | × |  |  |  |  |  |  |
| Ock and Rho, 2011 (95) [pig] |  | × | × |  | × |  |  | × |  |  |  |  |  |
| Heino et al., 2012 (39) [minipig] |  |  |  | × |  | × |  |  |  |  |  |  |  |
| Romanek et al., 2018 (98) [pig] |  | × |  |  |  | × |  | × |  |  |  |  |  |
| Mitchell et al., 2015 (32) [horse] | × | × |  |  | × | × |  |  |  |  |  |  |  |

Additional information 1 is a grid identifying which cell attributes each of the forty-one studies assessed. Of note each of the 41 studies may appear more than once depending on the attributes they assessed. Where a study undertook an assessment of a cellular attribute a cross is placed in the grid. Studies are arranged by species: human [chronologically and then alphabetically] and animals from most to least frequent species [chronologically and then alphabetically]). Column headers: Morphology; Morph. Viability; Via. Immunophenotyping; IP. Differentiation; Diff . Colony forming unit frequency; CFUF. Growth; Total growth. Metabolism; Met. Apoptosis; Apo. Attachment; Attach. Immunomodulation; Immuno. Paracrine; Para. Angiogenesis; Angio. Migration; Migr.
