# Supplementary material for: The impact of cryopreservation on bone marrow-derived mesenchymal stem cells: a systematic review
Source: J Transl Med. 2019 Nov 29;17:397. doi: 10.1186/s12967-019-02136-7 (PMC6883667; doi:10.1186/s12967-019-02136-7)
Supplement: Supplementary file 2 — Additional file 2. Tabulated information relating to the freezing details extracted from the relevant studies. It shows the details of the individual freezing protocols outlined in the 41 retained studies. The method of freezing is given in detail alongside the species information, the concentration and passage of cells at the point of cryopreservation and the process of thawing. These details are common to the results tables (Tables 1, 2, 3, 4, 5, 6, 7, 8, 9). [file 12967_2019_2136_MOESM2_ESM.docx]

Additional information 2. Tabulated information relating to the freezing details extracted from the relevant studies

| **Study** | **Species** | **Method of freezing** | **Concentration at freezing** | **Method of thawing** | **Passage number at freezing** |
| --- | --- | --- | --- | --- | --- |
| **Human** | | | | | |
| Bruder, Jaiswal and Haynesworth, 1997 (90) | Human | FBS with 10% DMSO in LN2 (24 hours) | NA | NA | NA |
| Hirose et al., 2004 (41) | Human | Cell Banker storage medium, cells cryopreserved at -150˚C (NA) | 5*10^5^ cells/mL | Cells were thawed in MEM-α supplemented with 15% FBS | P1 |
| Kotobuki et al., 2004 (35) | Human | Cell Banker medium, cryopreserved at -80˚C (NA) | 5*10^5^ cells/mL | NA | P1 |
| Kotobuki et al., 2005 (91) | Human | Cell Banker storage medium (ready-to-use storage medium), then cells stored sequentially: 10 min at 4˚C, 1h at -30˚C, 2-3 days at -80˚C then long-term storage at -152˚C (0.3-33.6 months) | 5*10^5^ cells/mL | NA | P1 |
| Haack-Sorensen et al., 2007 (19) | Human | EMEA-FBS 5% DMSO then LN2 (1 week) | 1*10^6^ cells/mL | NA | P2 |
| Xiang et al., 2007 (92) | Human | 30% serum-containing α-MEM with 10% DMSO, 4˚C for 10 min then cooled to -80˚C at 1˚C/min in a controlled-rate freezer then LN2 (12 months) | 1*10^5^ cells/mL | Thawed in a 37˚C water bath by shaking lightly for 1 or 2 min | P3 |
| Zhao et al., 2008 (93) | Human (with chronic myeloid leukaemia) | IMDM with 40% FCS and 10% DMSO at 4˚C, beaker with methanol in -70˚C freezer for 24h then then LN2 (3 or 6 months or 1 year) | 1*10^6^ cells/mL | 37˚C water bath for 2-4 min | P2-3 |
| Heng, 2009 (30) | Human | Culture medium with 10% DMSO and 0, 10 or 100 microM of Rho-associate kinase (ROCK) inhibitor Y-27632, cooling to -80˚C for 2h, then vapour phase of LN2 (1h) | 1.17*10^5^ cells/mL | Thawed in a 37˚C water bath | P5 |
| Liu et al., 2010 (28) | Human | 13 different freezing media tested with various combinations of different concentrations of serum, DMSO, PEG, trehalose and 1.2-Propanediol, equilibration of cells with freezing media at 4˚C for 10 minutes, -80˚C overnight then LN2 (min. 1 week) | 1*10^6^ cells/mL | Thawed in a 37˚C water bath, shaking gently for 2 min | N/A |
| Doan et al., 2012 (96) | Human | DMEM/F12 with 10% DMSO, incubation, 4˚C for 10 min, -20˚C for 1h, -80˚C for 1 day then LN2 (1 year) | 1*10^6^ cells/mL | In a water bath at 37˚C | P3 |
| François et al., 2012 (45) | Human | α-MEM with 30% FBS and 5% DMSO, -80˚C for 24h then LN2 (1 week) | NA | NA | Early passage |
| Ginis, Grinblat and Shirvan, 2012 (50) | Human | CryoStor-2, CryoStor-5, CryoStor-10 containing 2%, 5% and 10% DMSO respectively or conventional freezing medium (90% growth medium with 10% FCS, 30% bovine serum albumin and 10% DMSO), pre-cooling on ice for 10 min, slowly cooled to -5˚C, blast of chilling to -25˚C, quick return to -5˚C, cooling to -60˚C at a rate of 1˚C/min, cooling to -196˚C at a rate of -25˚C using programmable cell freezer then LN2 (about 1 month or 5 months) | 1*10^6^ cells/mL | Thawed fast in a 37˚C water bath with gentle agitation | P2-4 |
| Mamidi et al., 2012 (33) | Human | 90%FBS with 10% DMSO, programmable slow freezing unit then vapour phase of LN2 (long-term storage) | 3*10^6^ cells/2mL vial | Thawed in a 37˚C water bath, shaking gently for 1-2 min | P3 and then characterized at P4-6 (with another freezing at passage 4) |
| Matsumura et al., 2013 (26) | Human | COOH-PLLs 7.5% (w/w) at pH of 7.4 OR 10% DMSO in DMEM without FBS, -80˚C freezer (1 week or 24 months) | 1*10^6^ cells/mL | Thawed in a 37˚C water bath with gentle shaking | P3-5 |
| Chinnadurai et al., 2014 (20) | Human | Freezing media, -80˚C then LN2 (NA) | 5*10^6^ cells/mL | Quickly thawed (1-2 min) | P3-5 |
| Holubova et al., 2014 (68) | Human | 60% α-MEM medium with 30% pHPL and 10% DMSO, programmable controlled rate freezer at rate 1˚C/min to -80˚C then LN2 (1,3,6,7 and 8 months) | 1*10^6^ cells/mL | NA | P3 |
| Kumazawa et al., 2014 (37) | Human | CELLBANKER storage solution, cells stored at -80˚C (10 or more years) | NA | Cells thawed at room temperature | P3 |
| Moll et al., 2014 (38) | Human | 4 ˚C human blood type AB plasma containing 10% DMSO, frozen to -80˚C using rate-controlled cell freezing device (NA) | 1-2*10^6^ cells/mL | NA | P2-4 |
| Verdanova, Pytlik and Kalbacova, 2014 (25) | Human | 15 different freezing solutions containing various concentrations of DMSO (0, 1, 5, 10 and 100%) in the presence or absence of sericin at 1 or 5%, cooling to -80˚C at a rate 1˚C/min in a CoolCell container then LN2 (72 hours) | 1.4*10^5^ cells/mL | In a 37 ˚C water bath as quickly as possible | P1-3 |
| Al-Saqi et al., 2015 (65) | Human | 10%DMSO in Mesencult-XF or STEM-CELLBANKER at 4˚C, cryovials on ice then moved to -80˚C with a cooling rate -1˚C/min for 24h then then LN2 (NA) | 0.5-1*10^6^ cells/mL | Thawed in a 37˚C water bath for 1 or 2 min | P3 |
| Luetzkendorf et al., 2015 (40) | Human | 5% human albumin and 10% DMSO, automatized process in a programmable freezer then LN2 (21-51 days) | 1.8*10^8^ in cryopreservation bags | Thawed at | P3-4 |
| Pollock et al., 2015 (66) | Human | 60% plasmylate A, 20% of 25% HAS and 20% DMSO (Final concentration of DMSO was 10% by volume), controlled rate freezer then LN2 (30-45 days) | 1-10*10^6^ cells/mL | Thawed quickly in a 37˚C | P1-6 |
| Chinnadurai et al., 2016 (67) | Human | IFNɣ, caspase inhibitor Z-VAD-FMK or 3-Methyl Adenine pre-licensing 48 hours prior to cryopreservation, 5% human serum albumin, 5%, 20%, 40%, 90% hPL in aMEM with 10% DMSO OR CryoSOfree DMSO-free cryopreservation medium, cooling rate 1˚C/min then step-down freezing using a 7-step program in CryoMed controlled-rate freezer then LN2 (NA) | 5-10*10^6^ cells/mL | In a 37 ˚C water bath for 1 min | P2-6 |
| Gramlich et al., 2016 (18) | Human | CryoStor CS5 media, -80֯ C for 90 minutes then vapour phase of LN2 (7-30 days) | 1*10^6^ cells per mL | In a 37 ˚C water bath | P3-5 |
| Lechanteur et al., 2016 (34) | Human | 40% PBS + 40% of HSA solution (20%) + 20% DMSO added under agitation at 4˚C, automated cryofreezer with a 9-step program to -160˚C then vapour phase of LN2 (NA) | 2*10^6^ cells/mL | Freezing bag is protected in sterile plastic bag and thawed in a37˚C water bath for a few min | P3 |
| Yuan et al., 2016 (51) | Human (BM-MSC engineered to express TRAIL) | 5% DMSO, 30% FBS in alpha-MEM OR human albumin with 0.5-20% DMSO, isopropanol freezing box overnight in, -80˚C freezer then LN2 (1-3 weeks) | 1*10^6^ cells/mL OR 5*10^6^ cells/mL OR 10*10^6^ cells/mL | In a water bath at 37˚C with gentle shake for 2 min | P5 |
| **Other Species** | | | | | |
| Carvalho et al., 2008 (44) | Rat | DMEM with 10% FBS and 5% DMSO, cells incubate at room temperature for 15 min then vials cooled at 3˚C/min, 5˚C/min, 10˚C/min during 15, 45, 10 min respectively until -80˚C using programmable freezing device then LN2 (1 month) | 1*10^7^ cells/mL | Thawed in a 37˚C water bath with constant gentle shaking | Frozen down after 4 weeks in culture |
| Liu et al., 2011 (29) | Rat, mouse and calf | 14 different freezing solutions tested with various combinations of different concentrations of serum, DMSO, PEG, trehalose and 1.2-Propanediol, equilibration for 15 min at 4˚C, -80˚C overnight then LN2 (min. 1 week) | 1*10^6^ cells/mL | Thawed in a 37˚C water bath with gentle shaking for 2 min | NA |
| Naaldijk et al., 2012 (27) | Rat | Cryoprotectant consisted of hydroxyethyl starches of different mean molecular weights [MW= 109, 209, 309, 409, 509, 609 kDa] and/or DMSO, then cells were frozen according to one of seven different freezing protocols (NA) | 1*10^5^ cells/0.5mL | Thawed in a 37˚C water bath | P1-3 |
| Davies et al., 2014 (42) | Rat | 10% DMSO in 90% FBS, then vials incubated for 1h at 4˚C, 2h at -20˚C, overnight at -80˚C then LN2 (NA) | 1*10^6^ cells/mL | Thawing in a 37˚C RS Galaxy S + incubator for about 5 min | P1 |
| Renzi et al., 2012 (31) | Sheep, horse and rat | 13 different freezing media tested with various combinations of different concentrations of FBS, DMSO, Trehalose, hydroxyethyl starch, bovine serum albumin and Caspase inhibitor z-VAD-fmk, 4˚C for 60 min, gradual reduction of temperature -1˚C/min to -40˚C, -10˚C/min to -70˚C in a controlled rate freezer then vapour phase of LN2 (5 days) | 1*10^6^ cells/mL | Thawed in a 37˚C water bath | P4 |
| Li et al., 2009 (94) | Dog | DMEM with 10% FBS and 10% DMSO, 4˚C for 1h, -20˚C for 2h, -80˚C for 10.5h then LN2 (1 month) | 1*10^6^ cells/mL | Thawed at 37˚C | P4 |
| Zhu et al., 2013 (46) | Dog | DMEM containing 10% FBS and 10% DMSO, 4˚C for 1h, -20˚C for 2h, -80˚C for 10.5h then LN2 (3 years) | 1*10^6^ cells/mL | Thawed in at 37˚C | P4 |
| Edamura et al., 2014 (36) | Dog | Cryoprotectant solution with or without 10% DMSO and 10% FBS, biofreezing vessel at -80˚C in a freezer (7 days) | 1*10^6^ cells/mL | Thawed in a 37˚C water bath for 1 min | P1 |
| Tokumoto et al., 2008 (48) | Monkey | Cell Banker storage medium (serum free), -80˚C for 24h then a deep freezer at -150˚C (10 days) | 1*10^5^ cells/mL | Quickly thawed in a 37˚C water bath | NA |
| Nitsch et al., 2014 (97) | Monkey | Freezing medium containing 0,1,5,10 or 15% DMSO (v/v), controlled rate freezer using an optimised freezing rate then -150˚C freezer (1 week) | 1*10^6^ cells/mL | In a 37 ˚C water bath | P9 |
| Lauterboeck et al., 2016 (49) | Monkey | Three different freezing solutions tested (2 of them xeno-free) containing different concentrations of DMEM, DMSO and/or FBS, methylcellulose, poloxamer-188, α-tocopherol, cell suspension equilibrated for 10, 30 or 60 min then placed in controlled rate freezer using one-step freezing protocol or two-step freezing protocol then -150˚C (at least 24h) | 1*10^6^ cells/mL | In a 37 ˚C water bath for 90 seconds | NA |
| Ock and Rho, 2011 (95) | Pig | ADMEM solution supplemented with 10% FBS and 1% penicillin-streptomycin with 40%, 20% or 10% DMSO, controlled rate programmable freezing device at -1˚C/min from 25˚C to -80˚C then then LN2 (< 1 month) | 2*10^6^ cells/mL | In a 37 ˚C water bath for 1 min | P5 |
| Heino et al., 2012 (39) | Minipig | 90% FBS and 10% DMSO, slowly frozen in isopropanol chamber at -70˚C (11 weeks) | NA | Quickly thawed | P1 |
| Romanek et al., 2018 (98) | Pig (BM-MSC treated with a high hydrostatic pressure (HHP) before freezing) | 10% DMSO, 2h at -20˚C then LN2 (up to 4 weeks) | NA | 37˚C water bath with gentle shaking | NA |
| Mitchell et al., 2015 (32) | Horse | Six different freezing solutions tested (20% serum [autologous equine serum, commercial equine serum or FBS], 10% DMSO and 70% media OR 95% serum and 5% DMSO), -80˚C freezer for 24h then liquid phase of LN2 (2-5 days) | 10*10^6^ cells/mL | In a 35˚C water bath with gentle agitation | P3-6 |

Additional information 2 shows the details of the individual freezing protocols outlined in the 41 retained studies. The method of freezing is given in detail alongside the species information, the concentration and passage of cells at the point of cryopreservation and the process of thawing. These details are common to the results tables (Tables 1 to 9).
